# Activity-Related Dyspnoea in Chronic Pulmonary Diseases: New Mechanistic Insights

**Published:** 2017

**Authors:** Denis E. O’Donnell

Activity-related dyspnoea is the most common symptom of patients with chronic lung diseases and underpins perceived poor health status. Our understanding of the nature and source of dyspnoea continues to grow but successful amelioration of this distressing symptom can remain elusive, especially in those with advanced lung diseases.

According to Norman Jones, the great Canadian physiologist, “breathlessness can be seen to result from an imbalance between the demand for breathing and the ability to achieve the demand.” Indeed, in most clinical situations where patients report severe dyspnoea, ventilatory demand-capacity imbalance is present. Thus, in patients with chronic lung conditions, ventilatory demand reaches or exceeds maximal ventilatory capacity (MVC) during physical exertion. Similarly, the ratio of respiratory muscle effort (measured by esophageal manometry) to maximal possible respiratory effort is increased at a given work rate or ventilation in patients with lung disease versus healthy controls.

## Dyspnoea and increased inspiratory neural drive (IND)

Increased IND (compared with normal) is a common final pathway in dyspnoea causation in patients with chronic lung diseases during activity. The motor output of respiratory centers in the medulla and cortex cannot currently be measured directly. Tidal measurements of esophageal pressure and diaphragmatic electromyography (both expressed relative to maximum) provide indirect measures of motor command output, and are uniformly amplified in patients with lung disease compared to healthy individuals. The magnitude of IND during exercise is mainly determined by the CO_2_ output (VCO_2_) reflecting the metabolic requirement of the task. An additional determinant in patients with lung diseases is the extent of wasted ventilation [dead space (VD)] and the regulated arterial CO_2_ set-point. In most chronic lung diseases [COPD, interstitial lung disease (ILD), pulmonary arterial hypertension], the VD component of the tidal breath is abnormally high reflecting relatively reduced pulmonary blood perfusion of alveolar units with preserved or increased ventilation. This inefficiency of CO_2_ elimination by the diseased lungs results in increased chemostimulation of medullary centers and consequent increased IND. It is thought (based on animal studies) that sensory information about increased IND arising from the medulla (and motor cortex) is directly conveyed to the somato-sensory cortex where it is perceived as increased sense of respiratory muscle effort.

## Abnormal respiratory mechanics and dyspnoea

In healthy individuals during spontaneous breathing, tidal volume (VT) is positioned in the linear portion of the respiratory system’s pressure-volume (PV) relaxation curve. Even at high exercise intensities the expanding VT remains within this linear portion of the PV curve where the force-velocity and length-tension properties of the respiratory muscles are optimized. By contrast, in COPD and ILD inspiratory capacity (IC) is reduced indicating close proximity of VT to total lung capacity (TLC) and the “stiff” upper reaches of the PV curve, where the muscles of the respiratory system become overloaded (elastic loading) and functionally weakened. In this scenario of “high-end mechanics”, critical mechanical limits on VT expansion are in place despite near maximal IND. The growing disparity between increasing IND and VT after it has reached a plateau has been termed neuromechanical dissociation (NMD). We have postulated that NMD contributes to perceived “unsatisfied inspiration” – a distressing qualitative dimension of dyspnoea common in both obstructive and restrictive lung diseases, which is rarely reported in healthy individuals.

The neurophysiological construct described above provides a practical basis for an approach to the alleviation of dyspnoea in individual patients with chronic lung diseases. Thus, treatment is primarily directed towards reducing IND (e.g., reducing VCO_2_ or metabolic acidosis), improving mechanics (e.g., increasing IC) or modifying the affective aspect of dyspnoea (e.g., counseling, sedation).

